# Technologies for Fabricating Large-Size Diffraction Gratings

**DOI:** 10.3390/s25071990

**Published:** 2025-03-22

**Authors:** Changfeng Shao, Xinghui Li

**Affiliations:** Tsinghua Shenzhen International Graduate School, Tsinghua University, Shenzhen 518055, China; scf23@mails.tsinghua.edu.cn

**Keywords:** large diffraction gratings, tiled gratings, grating ruling engines, laser interference lithography, fringe-locking technologies, optical mosaic gratings, scanning beam interference lithography

## Abstract

Large-size diffraction gratings have played an important role in modern scientific fields such as inertial confinement fusion, large-aperture astronomical telescopes, and high-precision immersion lithography machines with long-stroke displacement stages. However, due to the large size and high accuracy requirements of gratings, and considering the need for high efficiency and low cost, the fabrication of large gratings is extremely difficult. This paper reviews the fabrication technologies for large diffraction gratings, including grating tiling technology, grating ruling technology, single-exposure lithography, optical mosaic grating technology, and scanning beam interference lithography. It introduces the basic principles, representative research, and research progress of these technologies, analyzes their advantages and current problems, and provides reference for the development and optimization of the fabrication technologies of large diffraction gratings.

## 1. Introduction

Diffraction grating is an important optical element, which contains a periodic spatial structure, such as parallel slits of equal spacing and an array of micro-nano patterns. Due to these periodic spatial structures, diffraction gratings can periodically modulate the amplitude or phase of incident light [1], thus playing an important role in various scientific research and industrial fields [2,3,4,5], such as spectral analysis [6,7,8,9], precision measurement [10,11,12,13,14], optical communication [15,16,17,18], and LiDAR [19,20,21].

**Figure 1:**
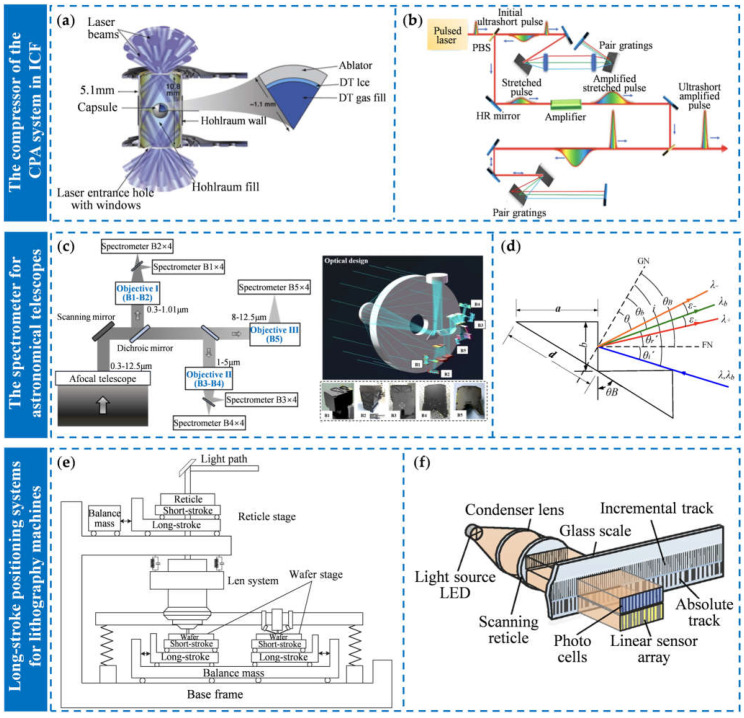
The applications of large-scale gratings: (**a**) the schematic of NIF ignition target [55]; (**b**) the scheme of chirped pulse amplification [56]; (**c**) the spectrometer for astronomical telescopes [57]; (**d**) the diffraction schematic of the echelle grating [58]; (**e**) the schematic of a lithography machine [59]; (**f**) an absolute grating encoder with a single M-code absolute track and an incremental track [60].

In recent years, driven by the demands of scientific research and engineering, diffraction gratings have developed towards larger sizes, smaller periods, and higher accuracy. Among them, large-size gratings with the meter-scale size have played a key role in numerous technical domains. Figure 1 shows the several applications of large-size diffraction gratings. The generation of high-energy laser used in inertial confinement fusion (ICF) relies on the high-power chirped-pulse amplification system (CPA) [22,23,24,25,26,27,28], as shown in Figure 1a,b. The amplification system utilizes the dispersion of the diffraction gratings to compress the amplified pulsed laser, which requires the diffraction grating to withstand an extremely high-power pulsed laser. Therefore, to provide more energy at the same damage threshold, the diffraction gratings are required to have a meter-scale size and high-quality diffraction wavefronts [29,30,31,32]. In large-aperture astronomical telescopes, to improve the resolution and signal-to-noise ratio of spectral analysis, the size of diffraction gratings is required to be in the meter scale [33,34,35], as shown in Figure 1c,d. In high-precision lithography machines, laser interferometers or grating encoders are usually used to achieve precise positioning of the wafer stage [36,37,38,39]. Compared to laser interferometers, grating encoders are more suitable for multi-degree-of-freedom measurement and have higher environmental robustness [40,41,42,43]. Therefore, the grating encoders have replaced laser interferometers in some lithography machines [44,45,46,47]. In recent years, the travel range of the wafer stage of immersion lithography machines has been required to be more than 300 mm [48,49,50]. Therefore, grating encoders in the measurement system needs to use large-size two-dimensional gratings [51,52,53,54], as shown in Figure 1e,f.

For the fabrication of large diffraction gratings, while expanding the grating size and ensuring high processing accuracy, it is also necessary to consider the efficiency and cost. Therefore, the fabrication of large-size gratings is difficult, and has always been a research hotspot. This paper introduces several fabrication technologies for large-size diffraction gratings, including their basic principles, representative research, latest achievements, and research progress, and analyzes the advantages and existing problems of these technologies. This paper consists of the following six sections. Section 1: introduction; Section 2: main fabrication technologies of diffraction gratings. The main content includes the advantages and disadvantages of the grating ruling technology, electron beam lithography, projection lithography, nanoimprint lithography, and holographic laser interference lithography; Section 3: the fabrication of large-size gratings based on the grating tiling technology. The main content includes grating tiling error theories (Section 3.1), grating tiling devices, and tiling stability control (Section 3.2); Section 4: the fabrication of large-size gratings based on the grating ruling technology; Section 5: the fabrication of large-size gratings based on laser interference lithography. The main content includes the fringe locking technology (Section 5.1), single-exposure lithography (Section 5.2), mosaic exposure lithography (Section 5.3), and scanning exposure lithography (Section 5.4); Section 6: conclusion and prospects.

## 2. Main Fabrication Technologies of Diffraction Gratings

**Figure 2:**
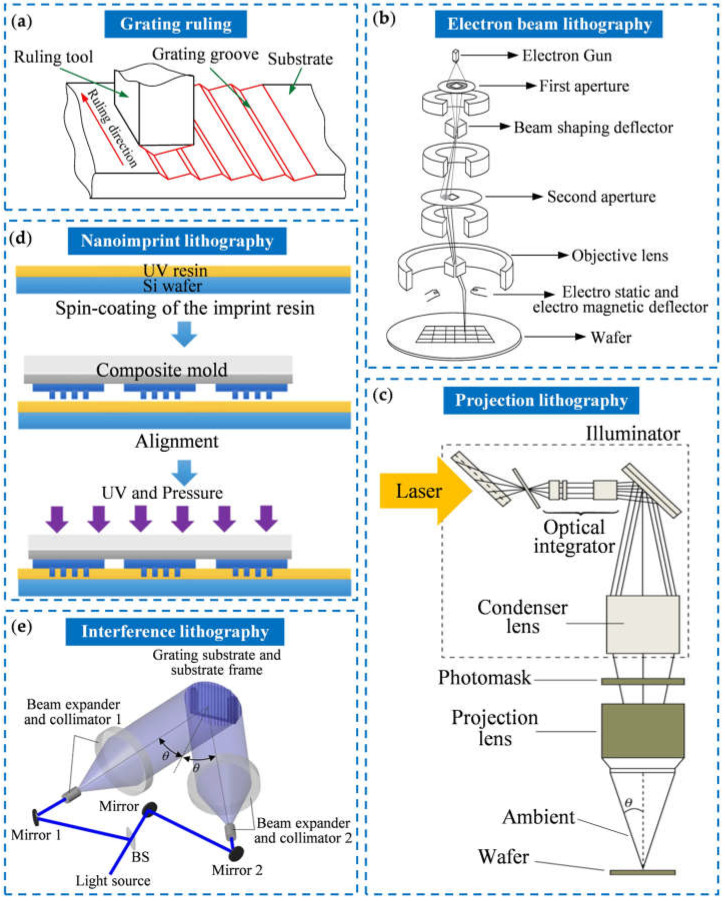
The fabrication technology of diffraction gratings: (**a**) the schematic view of a chisel-edge ruling tool and the grating ruling process [81]; (**b**) the electron beam lithography system [82]; (**c**) the scheme of the nanoimprinting and etching process [83]; (**d**) the schematic of the optical projection system [84]; (**e**) optical configurations for the laser interference lithography based on the division of amplitude method [85].

The fabrication technologies of diffraction gratings mainly include the grating ruling technology [61,62,63,64], electron beam lithography [65,66,67,68], projection lithography [69,70,71,72], nanoimprint lithography [73,74,75], and holographic laser interference lithography [76,77,78]. Figure 2 shows these fabrication technologies of diffraction gratings. For the manufacturing of large-size gratings, it is necessary to consider the processing accuracy, maximum processing size, processing efficiency, and the cost required [79,80].

The grating ruling technology is the earliest fabrication technology of diffraction gratings. The principle of grating ruling is to use diamond cutting tools to rule a series of equidistant groove lines on the surface of the substrate, as shown in Figure 2a. In the 19th century, scientists such as Fraunhofer, L. M. Rutherfurd, and H. A. Rowland designed grating ruling engines to fabricate gratings [86]. Over the next hundred years, researchers from various countries have successively improved grating ruling engines, using more precise carriages and servo control systems to reduce ruling errors. However, in the fabrication of large-size gratings, due to the long stroke of diamond cutting tools, the tools are prone to wear, thus resulting in variations in grating groove shapes and a decrease in diffraction efficiency. In addition, during the processing, temperature changes and vibrations in the environment also have a significant impact on the accuracy of ruling engines. When the number of groove lines increases, the ruling error of each groove line accumulates to a certain extent, causing ghost lines and stray light [87,88]. Therefore, if the grating ruling technology is used to fabricate large-size diffraction gratings, it is necessary to consider issues such as ruling tool life, environmental factors, and the overall accuracy of the ruling engine.

The principle of electron beam lithography (EBL) is to use an extremely small-aperture electron beam to draw a grating pattern on the photoresist above the substrate surface through scanning exposure and then complete the fabrication of gratings through development and etching. Figure 2b shows the principle of EBL. Due to the small aperture of the electron beam, EBL has extremely high accuracy and can draw any plane pattern on the photoresist [89,90]. However, the speed of EBL is very slow, resulting in low processing efficiency and high costs. Therefore, EBL is not suitable for the fabrication of large-size gratings [91].

The principle of projection lithography is to use the lithography machine to transfer the grating pattern from the mask to the photoresist on the substrate through exposure, as shown in Figure 2c. Projection lithography is relatively mature, with high processing efficiency and relatively small errors [92]. However, the weight and thickness of the substrates of large-size grating are much greater than wafers. The two-dimensional precision stage of a lithography machine cannot carry such a large-size substrate, so it is difficult for a lithography machine to process large substrates. In addition, when using the lithography machine to fabricate large-size gratings, it is necessary to process the substrate in different areas, which may cause the phase of the grating to be discontinuous. Therefore, projection lithography is rarely applied in the fabrication of large-size gratings.

Nanoimprint lithography is a new micro-nano fabrication technology that has been applied in the field of grating fabrication [93]. The principle of nanoimprinting is to transfer the pattern from the mold to the photoresist on the substrate surface through the imprint process, as shown in Figure 2d. Nanoimprint lithography has high processing resolution, low cost, and high efficiency, and has a great prospect in the field of micro-nano fabrication [94,95]. However, for the fabrication of large-size gratings, the pressure between the mold and the substrate may vary in different areas during the imprint process, resulting in a decrease in the replication accuracy of the mold pattern and the consistency of the grating groove depth. In addition, the fabrication of large-size and high-accuracy imprint molds is also difficult. Therefore, for the fabrication of large-size gratings, nanoimprint lithography still needs further development.

Laser interference lithography (LIL), also known as the interference exposure method, works by superimposing two coherent beams with identical wavelength to generate a periodic interference exposure field, and then using the photoresist on the substrate surface to record periodic interference fringe patterns [96]. Figure 2e shows the principle of LIL. The formula for the grating period g is defined by Equation (1), which is as follows:

(1) $$g=\frac{\lambda }{2\sin\theta }$$

where *λ* represents the wavelength, and *θ* is the incident angle [97]. By changing the wavelength and incident angle, the minimum period of the grating can reach the sub-micron level. LIL has the advantages of high processing efficiency, low cost, and no ghost lines, and the ability to fabricate small-period gratings [98]. In addition, LIL has a relatively large exposure aperture, which can be further expanded through optical mosaic methods [99]. Therefore, laser interference lithography is more suitable for the fabrication of large-size gratings [100]. Table 1 shows the advantages and disadvantages of these fabrication technologies.

## 3. Fabrication of Large-Size Gratings Based on the Grating Tiling Technology

There are three main methods for the fabrication of large-size diffraction gratings, namely grating tiling technology, grating ruling technology, and holographic laser interference lithography. Among them, grating ruling and laser interference lithography are capable of fabricating single large-size gratings. In contrast, grating tiling technology cannot fabricate single large gratings as it only utilizes an array of smaller gratings to substitute for a large grating and replicate the function of the large-size grating.

### 3.1. Grating Tiling Error Theories

**Figure 3:**
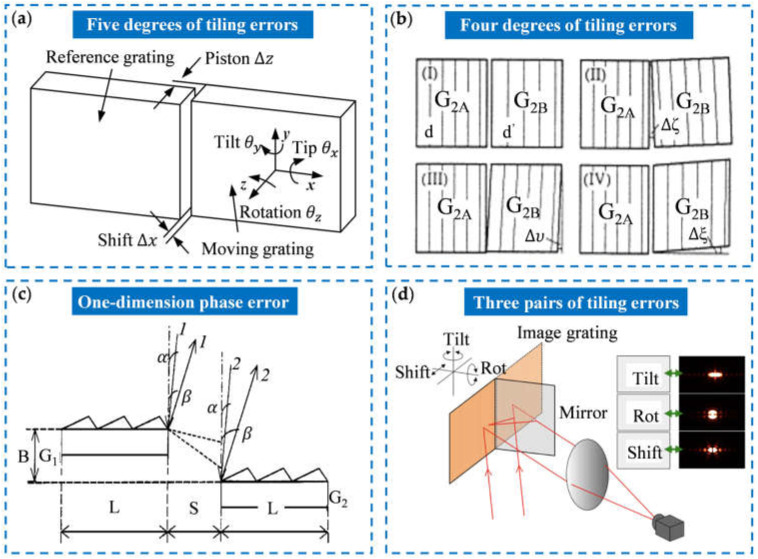
Grating tiling error models: (**a**) five degrees of freedom between the two adjacent diffraction gratings [104]; (**b**) four degrees of tiling errors in the tiled gratings [105]; (**c**) phase matching of the diffraction gratings [106]; (**d**) motion tiling errors within an object-image-grating self-tiling [109].

The principle of grating tiling technology is to tile several relatively small gratings through mechanical devices and adjust the position and posture of each small grating to control the overall diffraction wavefront distortion within the error range, thus substituting for a single large-size grating [101,102,103]. In the tiled gratings, the five degrees of tiling freedom between adjacent sub-gratings are shown in Figure 3a. Usually, one of the gratings is selected as a fixed reference grating, and the other is the moving grating. Since the tiling accuracy of these degrees of freedom cannot be controlled absolutely, the tiling errors will be generated during tiling, which will reduce the diffraction wavefront quality of the tiled grating. Therefore, researchers have performed a lot of research on the theory of grating tiling error and established various error models.

Tiling errors include lateral shift ∆x, longitudinal piston ∆z, angular tip $\theta_{x}$, angular tilt $\theta_{y}$, linear rotation $\theta_{z}$, and grating period difference ∆d [104]. In 1998, Zhang et al. proposed a theoretical model of a tiled grating compressor for a chirped-pulse amplification system and analyzed the effects of various tiled errors on the far-field time domain of the compressed pulse [105]. The error model is shown in Figure 3b. The analysis shows that under the criterion that the stretch in the compressed pulse is kept below 25%, the ratio of groove-width difference should be less than $1.6\times 10^{-4}$, the groove line parallelism error should be less than 0.3 mrad, and the two types of planar errors ∆ζ and ∆ξ should be less than 0.18 mrad and 0.015 mrad, respectively. In 2000, Zhao established a phase error model for one-dimensional tiled gratings and analyzed the effects of various tiling errors on the phase of the sub-grating diffraction wavefront [106]. Figure 3c shows the tiling model of a one-dimensional grating. When the grating horizontal spacing is an integer multiple of the grating period and the grating vertical spacing is 0, the phases of the diffraction wavefronts of each sub-grating are consistent. In 2004, Harimoto analyzed the effect of tiling errors on the far-field pattern of monochromatic light and provided a theoretical basis for the tiling error detection and alignment based on the far-field pattern [107]. Kessler et al. from the University of Rochester proposed the principle of tiling error pairs [108], which reduces the six-dimensional tiling errors to three-dimensional error pairs, reducing the difficulty of error adjustment and stability control of tiled gratings. In 2015, Li et al. from Suzhou University proposed a new idea to adjust tiling errors, as shown in Figure 3d [109]. They used a mirror to replace a grating, so that the grating was tiled with its image formed in the mirror, so only three-dimensional tiled errors needed to be adjusted.

### 3.2. Grating Tiling Devices and Stability Control

**Figure 4:**
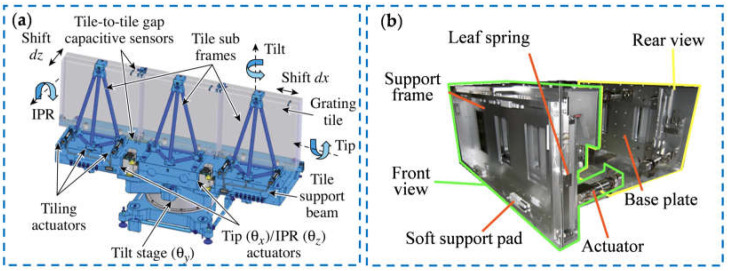
The grating tiling device: (**a**) the grating tiling system of Rochester University [110]; (**b**) the grating tiling device of Osaka University [111].

Based on the tiling error theories, researchers from various countries have conducted experimental research on grating tiling devices and stability control. In 2007, Qiao et al. from the University of Rochester designed a tiling device, as shown in Figure 4a [110], which tiled three gratings with a size of 0.47 m into a large grating with a size of 1.41 m. Each small grating was fixed by a support frame, and the tetrahedral truss was used to ensure the accuracy and stability of the grating tiling device. The grating tiling device could adjust the five freedom degrees of the moving grating through an intermediate turntable, two electric linear actuators, and three piezoelectric ceramic actuators, and could reach a translation accuracy of 130 nm and a rotation accuracy of 0.2 μrad. In 2008, researchers from Osaka University designed a grating tiling device, as shown in Figure 4b, for the FIREX-1 project [111], which tiled two gratings into a large-size grating of 920 mm × 410 mm. The actuation unit of the device could achieve precise adjustment of the position and posture of the moving grating. The calculation results showed that the RMS value of the surface deformation of the tiled grating is less than 0.05λ. The CEA CESTA in France designed a grating tiling device to obtain several gratings with a size of 450 mm × 420 mm [112,113]. They adjusted the tiling errors of moving grating through a mechanism which consisted of a V-shaped slope and a steel ball, and added rib plates to improve the tiling stability. This tiling device could reach a translation accuracy of 50 nm and a rotation accuracy of 1 μrad. The University of Jena, Germany, also designed a tiling device [114]. They used high-precision piezoelectric actuators to drive the translation rotation stages, so that the translation error was less than 40 nm and the rotation error was less than 0.4 μrad. Zhang et al. from Chongqing University, China, also conducted experimental research on grating tiling [115,116]. In 2011, they designed a 2 × 2 tiling device [117]. This tiling device could tile four gratings with a size of 210 mm × 150 mm. The tiling accuracy was calculated to be 60 nm by measuring the grating dispersion focus. In 2015, they designed a tiling device using the truss, which could tile two gratings with a size of 420 mm × 210 mm [118]. This device used a structure combining a V-shaped slope and a steel ball to support the moving grating, and used a tetrahedral truss to improve the stability of the tiled grating. The adjustment unit used piezoelectric ceramics actuators, which could adjust the three freedom degrees of tiling error pairs accurately.

**Figure 5:**
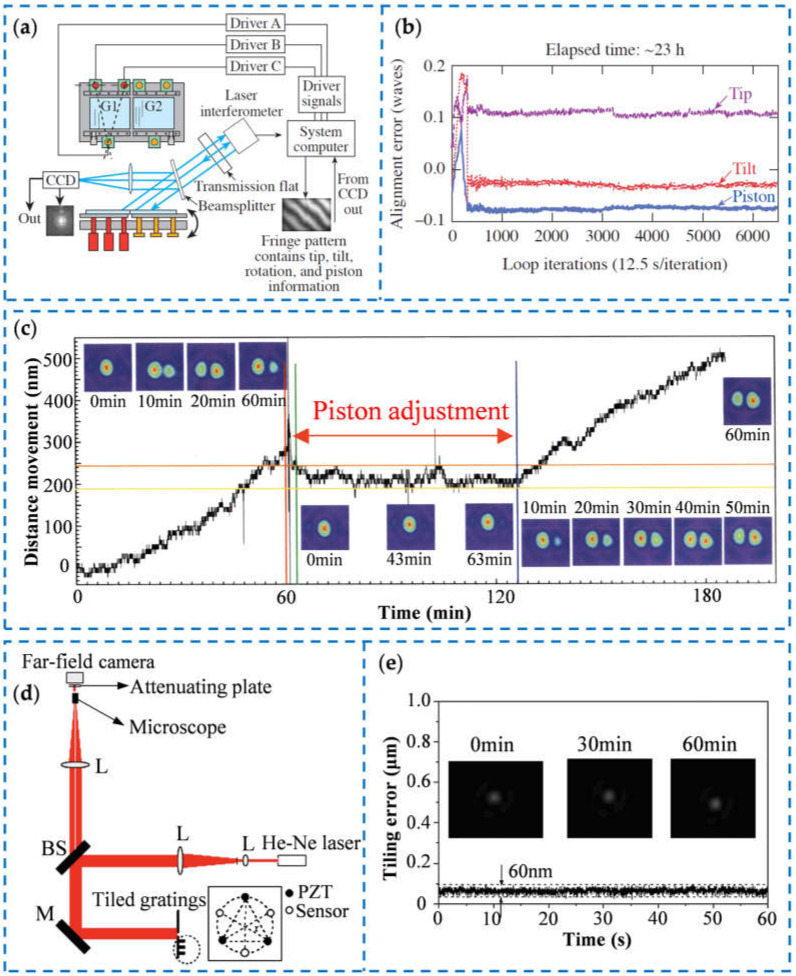
Stability control of the tiled gratings: (**a**) the system diagram of pose detection [120]; (**b**) the time stability of tiling errors [120]; (**c**) temporal evolution of displacement of one motion sensor [122]; (**d**) the schematic of focal spot measurement for different phase dispersions [117]; (**e**) the result of different focal spots within 1 h [117].

To obtain high-accuracy tiled gratings that can be used for a long time, it is necessary to detect and align the tiling errors in real time to ensure the stability of the tiled gratings [119]. At present, the position and posture deviation of the moving grating is generally detected by optical measurement or high-precision displacement sensors and then used as feedback to drive the high-precision displacement actuators to achieve closed-loop control of the grating pose. The Laser Energetics Laboratory of the University of Rochester used a CCD camera to monitor near-field interference fringes [120] and then analyzed the pose change of the moving grating through the interference fringes. Then, they used this pose change as the feedback of the closed-loop control system and used three piezoelectric actuators for error compensation to achieve long-term stability of the tiled grating. The detection system is shown in Figure 5a, and the relationship between the tiling error and time is shown in Figure 5b. The tip error, tilt error, and piston error were all controlled under 0.1λ within 23 h. The tiling device of Japan’s FIREX-1 project used a capacitive displacement sensor to monitor the pose change of the tiled grating [121,122], and maintained the stability of the tiled grating through the real-time adjustment of the piezoelectric actuators. Figure 5c shows the test results of the stability of the tiling gratings. In this way, the piston error could be less than 50 nm within 60 min. Zhang et al. from Chongqing University also used a capacitive micro-displacement sensor to detect the pose change of the moving grating [117], and developed a control method for the tiled grating pose based on fuzzy control technology. The optical measurement system is shown in Figure 5d. They collected the far-field pattern through a CCD camera, evaluated the control result through calculation, and then input it into the closed-loop control system as a feedback value to achieve long-term stability of the tiled grating. Figure 5e shows the drift of the far-field pattern. Within 60 min, the position and posture of the tiled grating was maintained at an accuracy of 60 nm. Yang et al. from the National University of Defense Technology also used a similar measurement system and used a CCD camera to detect the far-field pattern, obtaining a tiling device with an accuracy of 60 nm within 60 min [123,124].

The advantage of grating tiling technology is that the fabrication of small sub-gratings is relatively simple, low-cost, and high-quality. However, in practical applications, high-precision control and adjustment technology must be used continuously, and the stability of the support frame is required to be high. In addition, the seams between the sub-gratings will cause spectral and energy loss. Moreover, when the number of sub-gratings increases, the structure of the tiling device becomes more complicated, and it is difficult to ensure the long-term stability of the system. Therefore, the large-size gratings fabricated by grating tiling are not suitable for some application scenarios.

## 4. Fabrication of Large-Size Gratings Based on the Grating Ruling Technology

Grating ruling technology is one of the main processing methods for diffraction gratings and is the original and most mature large-size grating fabrication technology. In 1882, Rowland from Johns Hopkins University invented a grating ruling engine, which produced the world’s best diffraction grating at the time, with a width of 152 mm and a groove line density of more than 650 lines/mm [125]. During the ruling process, the grating blank made an indexing one-way motion on the blank carriage, and the diamond ruling tool made a reciprocating ruling motion in the direction which was perpendicular to the blank’s direction of motion. The Rowland engine used a purely mechanical ruling and positioning method. The positioning of the grating grooves depended on the mechanical transmission system with the lead screw. Therefore, the processing accuracy of the grating was subject to the processing and assembly accuracy of each component in the mechanical system. In the mid-20th century, Soviet researchers designed a moiré-controlled grating ruling engine based on the moiré-fringe interferometry [126]. The moiré-fringe control system can accurately position the carriage of the ruling engine, with a maximum ruling width of 300 mm, a maximum ruling density of 2400 lines/mm, and a relative ghost line intensity of less than 0.01% [127]. Later, due to the further development of control technology, researchers began to introduce servo control technology into the grating ruling, and the ruling engine was able to fabricate larger and better-quality diffraction gratings. At present, most of grating ruling engines in the world have adopted the Rowland engine’s working mode and servo control systems. On this basis, according to the different motion modes of the grating blank carriage, grating ruling engines are divided into three types: continuous, intermittent, and stop-and-go.

**Figure 6:**
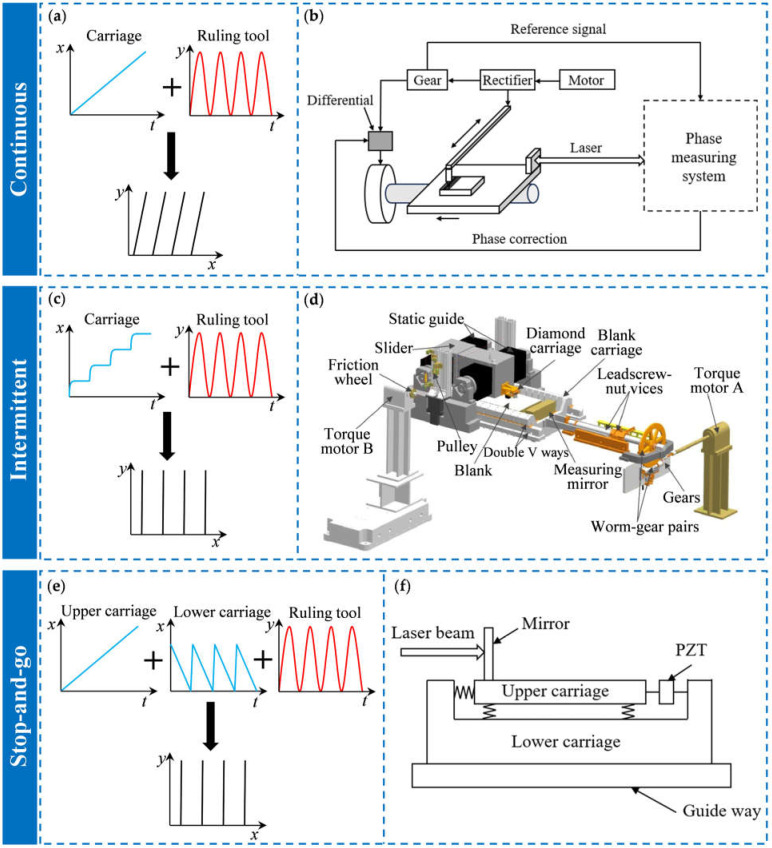
Three types of grating ruling engines: (**a**) the schematic diagram of the continuous ruling method; (**b**) the system of MIT-A ruling engine [129]; (**c**) the schematic diagram of the intermittent ruling method; (**d**) the schematic diagram of the CIOMP-6 ruling engine [132]; (**e**) the schematic diagram of the stop-and-go ruling method; (**f**) the schematic diagram of the Hitachi-4 blank carriage [133].

In the continuous ruling engine, the reciprocating motion of the ruling tool and the one-way motion of the grating blank are continuous and simultaneous, and their motion trajectories are synthesized to achieve grating ruling. Figure 6a shows the result of motion trajectory synthesis, and the groove lines are oblique lines with an equal distance. The most representative continuous ruling engines are the MIT-A, -B, and -C grating ruling engines. In 1955, Harrison from MIT first introduced the Michelson interferometer measurement system and servo control system into the grating ruling engine and developed the MIT-A ruling engine, which improved the processing accuracy and the quality of the ruled gratings [128,129]. The system structure of the MIT-A ruling engine is shown in Figure 6b. This system used a differential as a feedback actuator, with its input shaft connected to the phase-correction motor and its output shaft connected to the worm gear. During the ruling process, the displacement signal of the worktable was collected through the interferometer measurement system. Then, the displacement signal phase was compared with the reference phase from the gears to determine whether the worktable had a positioning error. When the measurement system detected that the worktable had a positioning error, the generated phase difference signal would drive the phase correction motor to make a small adjustment to the transmission ratio between the input shaft and the output shaft, resulting in it reducing the error. Harrison’s team also developed the MIT-B ruling engine based on the Moore’s No. 3 measuring machine [130]. This ruling engine used a V-shaped blank carriage to reduce the grating stray light caused by the irregular changes in the motion trajectory of the worktable. Moreover, it also reduced the swing angle and positioning errors of the worktable and produced a 210 mm × 410 mm high-quality echelle grating. In 1966, Harrison referred to the development experience of the MIT-A and MIT-B and used Moore’s No. 4 long-stroke measuring machine to develop a grating ruling engine with a larger ruling area, the MIT-C engine [131]. Harrison’s team used this ruling engine to produce several large-size echelle gratings of 350 mm × 450 mm, with a line density of 31.6 to 632 lines/mm. The resolution was 90% of the theoretical value, and no Rowland ghost lines were observed.

For the intermittent ruling engine, during the ruling process, the indexing system drives the worktable to move a grating period length and then stops. The diamond ruling tool falls, rules a line, and then returns to the starting position. These two processes are repeated continuously to achieve grating ruling, as shown in Figure 6c. The CIOMP series ruling engines designed by the Changchun Institute of Optics, Fine Mechanics, and Physics (CIOMP) adopt this intermittent ruling mode. In 2016, CIOMP developed the large-scale high-precision grating ruling engine CIOMP-6, with a maximum design ruling area of 420 mm × 520 mm [134,135]. The system is shown in Figure 6d. CIOMP-6 uses a new diamond-tool carriage system based on aerostatic guideways and a new blank carriage system with double piezoelectric actuators to reduce positioning errors. At the same time, they also proposed a servo control system with an optical measurement system with macro- and micro-positioning which can control the position of the diamond relative to the blank [136,137]. This made the processing accuracy of the ruling engine better than 4 nm. The diffraction efficiency of the ruled grating was close to the theoretical value, and the stray light was less than $10^{-5}$. No ghost lines were observed. The ruling engine has produced a high line density grating with 8000 lines/mm and an echelle grating with an area of 400 mm × 500 mm and a line density of 79 lines/mm [132].

Stop-and-go ruling is a ruling method developed by Hitachi. The indexing system of the stop-and-go ruling engine had a macro and micro two-carriage positioning system. The macro positioning carriage moved continuously, and the speed of the micro positioning carriage was equal to the speed of the macro positioning table but in the opposite direction. This allowed the grating blank to remain stationary during a single scribing process. The motion synthesis of the ruling system and the indexing system is shown in Figure 6e. In 1992, Hitachi developed a high-precision grating ruling engine, Hitachi-4, which adopted the stop-and-go motion mode. The lower carriage was a macro-positioning carriage, and the upper carriage was a micro-positioning carriage. A piezoelectric ceramic actuator was installed between the upper and lower carriages. The system is shown in Figure 6f. During the ruling process, the lower carriage drove the blank in a large range, and the upper carriage could make precise adjustments to its position and swing angle under the drive of the piezoelectric ceramic actuator, thereby improving the positioning accuracy. In this way, the positioning accuracy of the Hitachi-4 worktable was 5 nm, the maximum ruling area was 300 mm × 200 mm, and the maximum ruling density was 10,000 lines/mm [133]. Table 2 shows the advantages, disadvantages, and applicable scenarios of these three types of ruling engines.

In grating ruling, the shape and size of the ruled grating grooves are determined by the diamond tools [138,139,140]. Diamond tools can reach very high accuracy, so grating ruling technology is very suitable for the fabrication of large-size diffraction gratings with special groove shapes and high aspect ratios, such as large gratings in the infrared and near-infrared bands required for astronomical telescopes and echelle gratings with excellent spectroscopic capabilities. For example, Hyperfine’s grating ruling engines H-7 and H-8 were specially designed to fabricate the echelle gratings with a low groove density which are required for astronomical observations. Their theoretical maximum ruling areas were 609 mm × 1219 mm and 1219 mm × 1219 mm, respectively, and the processed substrate was aluminum alloy. The H-7 ruling engine fabricated the airborne infra-red echelle spectrometer (ARIES) grating for NASA-AMES, with a size of 254 mm × 1066.8 mm, a grating period of 980 μm, and a blaze angle of 76°, making it the largest single ruled grating in the world [141]. However, since the tool can only process one grating line each time, when the line density of the large-size grating is high, the processing efficiency of grating ruling is very low, and it takes weeks or even months to fabricate one grating [142]. During such a long time, the diamond tool may be severely worn, which will cause the shape of the grating grooves to change, affecting the performance of the grating. In addition, the existing ruling engines are unable to fabricate high-precision diffraction gratings with the meter scale and high line density. Therefore, grating ruling technology still needs to be further developed in terms of processing efficiency and the maximum processing size of the grating.

## 5. Fabrication of Large-Size Gratings Based on Laser Interference Lithography

With the advent of lasers, holographic laser interference lithography (LIL) has begun to be applied in the field of grating fabrication. Compared with traditional grating ruling technology, the gratings fabricated by LIL do not have the ghost lines and stray light in diffraction. In addition, LIL has a short processing time, high success rate, and large processing area. Its processing efficiency is much higher than that of grating ruling. Therefore, it is often used in the fabrication of large-size diffraction gratings. Fabrication technologies of large-size grating based on LIL are mainly divided into single-exposure lithography [143,144,145], mosaic exposure lithography [146,147,148,149], and scanning exposure lithography [150,151,152,153].

### 5.1. Fringe Locking Technology

**Figure 7:**
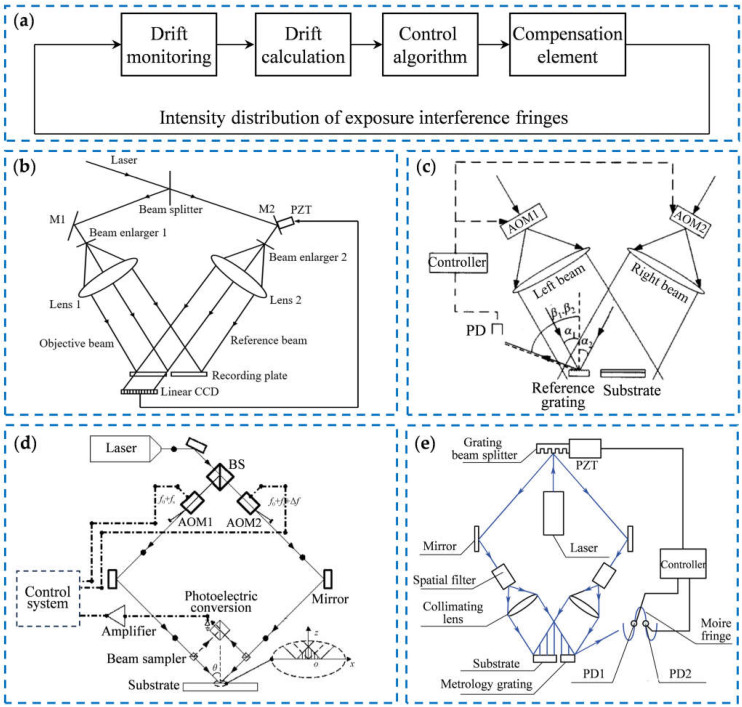
The fringe locking systems: (**a**) the block diagram of a usual fringe locking system; (**b**) the schematic of a phase-shift fringe-locking system [160]; (**c**) the schematic of a frequency-shift fringe-locking system [163]; (**d**) the schematic of scanning interference lithography machine and frequency-shift fringe-locking system [164]; (**e**) the schematic of a frequency-shift fringe-locking system with grating beam splitter [168].

Before introducing these three interference exposure lithography technologies, we first introduce the fringe locking technology [154,155,156,157]. Fringe locking technology is a control method that monitors the phase of the interference exposure field in real-time during the exposure process and uses compensation elements to keep the phase of the interference exposure field stationary relative to the substrate [158,159]. The block diagram of a typical fringe locking system is shown in Figure 7a. Fringe locking technology can achieve phase stability of the interference fringes during the exposure process, thereby improving the processing accuracy of the gratings, and plays an important role in single-exposure lithography, mosaic exposure lithography, and scanning exposure lithography in the fabrication of large-size gratings. According to the compensation method for fringe drift, fringe locking technology can be divided into phase-shift fringe locking and frequency-shift fringe locking.

The phase-shift fringe locking adjusts the optical path difference in the two interfering optical paths through the compensation element to change the relative phase of the interfering laser beams, thereby achieving the locking of the interference fringe phase. In 2009, Li et al. from Suzhou University proposed a fringe locking method based on reference grating’s moiré fringes and the mirrors driven by piezoelectric ceramic actuators [160]. The principle of the locking system is shown in Figure 7b. In this system, the −1 order diffractive light generated by the reference grating arranged in the exposure interference field interfered with another exposure beam to produce macroscopic reference fringes which were visible to the human eye. The reference fringes were monitored by a CCD camera, and the piezoelectric ceramic actuator was used to drive a mirror to change the optical path difference after calculating the drift, thereby achieving fringe-phase compensation. After using this system, the mean square error of the interference fringe drift value was lower than λ/60. In 2011, Zeng et al. from Tsinghua University proposed a self-referenced fringe-locking method based on latent grating for the fabrication of optical mosaic gratings [161]. Like the fringe-locking system of Suzhou University, this method also uses phase-shift fringe locking. They used an EMCCD camera to record the reference fringes produced by the latent grating and determined the magnitude and direction of the phase drift by calculating the light intensity at the sampling points of the fringe images in real-time. Then, they drove the PZT installed behind one of the mirrors to move along the normal direction, and achieved phase compensation by changing the optical path difference between the two paths. This method did not introduce external optical elements into the optical paths and was a completely self-referenced method, thus avoiding measurement errors caused by an external measurement system. However, due to the low diffraction efficiency of the latent grating, this locking system had high requirements for the sensitivity of the optical imaging device and the control of ambient stray light and could not achieve high-frequency real-time control.

The frequency-shift fringe-locking method uses an acousto-optic modulator (AOM) or the Doppler frequency shift effect to produce a frequency difference between the two exposure beams, thereby achieving the locking of the interference fringes. In 2014, Song et al. from the Changchun Institute of Optics, Fine Mechanics, and Physics proposed a frequency-shift fringe-locking system [162,163], as shown in Figure 7c. They fixed a measuring reference grating in the exposure area to generate reference fringes and used a photodiode (PD) to monitor the optical power in real-time to determine the phase drift. When phase drift occurred, the phase was compensated by controlling the carrier frequency of the AOM on the two beams. This method had a large adjustment range and a fast compensation speed and could make the phase drift of the exposure interference fringes less than 0.02 period. In 2015, Zhu et al. from Tsinghua University developed a homodyne frequency-shift fringe locking system for scanning exposure lithography systems [164], as shown in Figure 7d. The system used homodyne interferometry to measure the phase drift of the exposure interference fringe and uses the AOM to achieve frequency-shift fringe locking. Tests showed that the system had 1/25 interference fringe period accuracy. Subsequently, they also used the extended Kalman filter algorithm, LQG controller, and sliding mode control algorithm (SMC) to improve the performance of the fringe-locking system [165,166,167]. By introducing certain predictive information in the fringe-phase monitoring, the dynamic performance of the fringe-locking system was further improved, so that it achieved a locking performance better than the traditional PID control algorithm in high-frequency random noise. In 2017, the CIOMP team developed a new frequency-shift fringe locking method [168], as shown in Figure 7e. The system used a grating beam splitter to split the laser, which was driven by the PZT, and utilized the Doppler frequency difference in the moving grating to compensate for the exposure fringe-phase. Results showed that this fringe-locking system could make the phase drift of the exposure fringe less than 0.021 period.

For the commercial solutions of fringe locking systems, the representative companies are Odhner Holographics and PGL in the United States. Odhner Holographics launched an interference fringe-locking device, Stabilock II. The device used a photodetector to obtain the phase drift of the laser and used a galvo scanning system to compensate the phase. The fringe-locking device could reach a phase-locking accuracy of 0.05λ and a compensation range of ±5 μm, and had been applied in holographic lithography. The joint team of PGL and MIT launched a scanning beam interference lithography machine, Nanoruler II [169]. The fringe-locking system used the splitting of the exposure beam to generate a heterodyne interference signal, used a photodetector to monitor the phase drift of the interference signal, and then used an AOM to compensate the phase, which could achieve a phase-locking accuracy of 0.01λ.

### 5.2. Single-Exposure Lithography

The principle of the single-exposure lithography is to use a large collimating lens to expand the laser beam to the meter size, thereby obtaining a large exposure area, and then directly transfer the interference fringe pattern to the substrate through single exposure. The most representative institution for single-exposure lithography is the Lawrence Livermore National Laboratory (LLNL) in the United States. In 2001, LLNL adopted a dual-beam exposure method, using a 1.1 m diameter lens to collimate two Kr-ion laser beams which were made to interfere at an incident angle and irradiate the substrate [170]. The exposure area was a circular area with a diameter of 1 m, and the grating groove line density could reach 2000 lines/mm. In addition, due to the large size and weight of the grating substrate, the traditional spin coating method could not make the photoresist layer evenly distributed on the large-size substrate. Therefore, LLNL developed a meniscus coating machine specifically for coating large-size grating substrates, which can coat 1 m × 2 m grating substrates, and the peak-to-valley deviation of the photoresist film thickness was less than 5%. At the same time, LLNL also developed dedicated ion etching equipment that could etch meter-scale gratings. Using meter-size collimating lenses and the dedicated equipment, LLNL has produced a series of 910 mm × 450 mm multilayer dielectric film gratings for the National Ignition Facility (NIF) of the United States, with a groove line density of 1752 lines/mm, a diffraction efficiency of up to 97%, and a damage threshold of the order of J/${\mathrm{cm}}^{2}$ (pulse width 10 ps).

In 2017, to fabricate meter-scale gratings, Hong et al. from the University of Science and Technology of China developed meter-scale meniscus coating equipment and a scanning ion etching machine [171,172], which achieved the coating and etching of a meter-scale grating substrate. After coating, the overall film thickness error was less than 4%, and the film thickness uniformity was good, meeting the error requirements of photoresist film thickness on the substrate surface of large-size gratings. In 2024, the Shanghai Institute of Optics and Fine Mechanics used ultra-large aperture reflective single-exposure lithography to fabricate a 1620 mm × 1070 mm seamless pulse compression grating [173,174]. Its average diffraction efficiency was higher than 93% [175].

In single-exposure lithography, the substrate only needs to be exposed once and so the processing efficiency is relatively high, and the quality of the fabricated grating is also high, without producing ghost lines and stray light. However, single-exposure lithography requires large-size lenses to collimate the laser beam, so the maximum processing size of the grating is limited by the aperture of the collimating lens. It is very difficult to fabricate meter-scale collimating lenses, which require huge costs [176,177]. It is the main bottleneck of single-exposure lithography.

### 5.3. Mosaic Exposure Lithography

**Figure 8:**
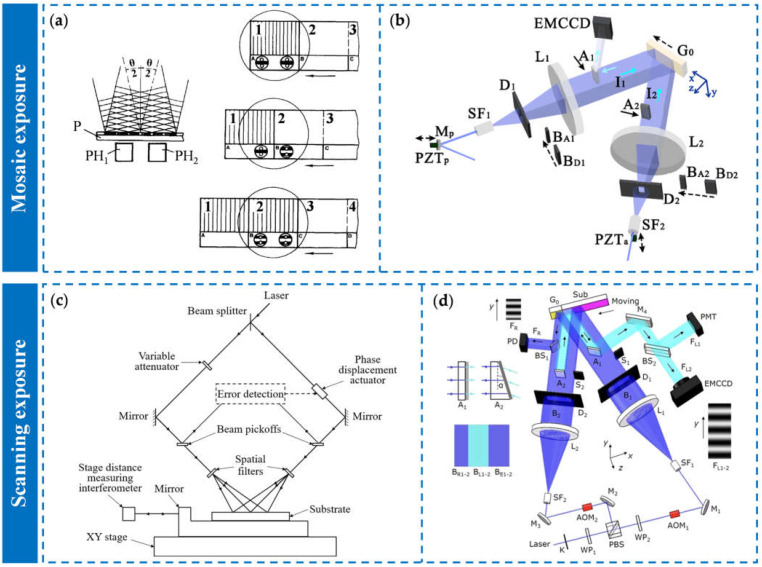
The fabrication technologies of the large-size gratings based on LIL: (**a**) the schematic of phase-synthesis method for mosaic gratings [178]; (**b**) the mosaic grating system based on latent reference grating [182]; (**c**) the system of scanning beam interference lithography of MIT [183]; (**d**) the device of broad-beam scanning beam interference lithography with self-referencing alignment [184].

Inspired by of grating tiling technology, researchers developed an optical mosaic exposure technology based on the holographic laser interference lithography. The principle of mosaic exposure lithography is to expose different areas of the grating substrate multiple times to expand the area of the grating and fabricate a large-size grating that exceeds the aperture of the collimating lens. In 1996, Turukhano et al. from the St. Petersburg Institute of Nuclear Physics in Russia proposed a mosaic exposure method to fabricate large-size long-diffraction gratings [178]. The mosaic method is shown in Figure 8a. Areas 1, 2, 3, and 4 were parts of a grating substrate. A, B, C, and D were several independent reference gratings, whose periods were equal to the period of the exposure interference fringe, and these reference gratings were fixed to the substrate. ${\mathrm{PH}}_{1}$ and ${\mathrm{PH}}_{2}$ were two photodetectors placed under the reference gratings to record the phase of the interference fringes generated by the reference gratings. The circular area was the exposure interference field. During the first exposure, ${\mathrm{PH}}_{1}$ recorded the phase of the fringes generated by the A reference grating as $\varphi_{1}$. After that, the substrate was moved by about half the aperture of the exposure interference field so that the phase recorded by ${\mathrm{PH}}_{1}$ was still $\varphi_{1}$. At this time, ${\mathrm{PH}}_{2}$ recorded the phase of the fringes generated by the B reference grating as $\varphi_{2}$. The substrate was moved so that the phase recorded by ${\mathrm{PH}}_{1}$ was $\varphi_{2}$, and then the second exposure was started. By this method, the phase difference between the grating lines in area 1 and the grating lines in area 2 was zero. By repeating the above operation, a grating with a size exceeding the exposure area aperture could be obtained. Turukhano tested the mosaic grating and found that the cumulative error of the grating lines was (0.03 + 0.4 L) μm, where L was the total length of the grating. However, Turukhano’s mosaic exposure method is only suitable for the fabrication of linearly sized gratings. In 2009, Li et al. from Suzhou University used the mosaic exposure method based on reference grating and fringe-locking technology to process an 80 mm × 110 mm diffraction grating with a groove line density of 1740 lines/mm and a diffraction wavefront error of less than 30 nm [179,180]. In 2016, a team from Suzhou University, University of Science and Technology of China, and Tsinghua University combined the advantages of the large-aperture exposure interference field and mosaic exposure method, and used a large-size lens with an aperture of 750 mm to fabricate a grating with a size of 1400 mm × 420 mm and a groove line density of 1700 lines/mm through mosaic exposure [181].

In the mosaic exposure method based on reference grating fringe locking, the relative drift between the reference grating and the substrate reduces mosaic accuracy [185]. Moreover, the size of the reference grating limits the maximum processing size of the grating. To solve these problems, researchers have proposed the mosaic exposure method based on latent grating fringe locking. In 1997, Napier et al. proposed a mosaic exposure method based on latent gratings to fabricate fiber Bragg gratings [186]. They used pulsed laser for exposure and photoresist-insensitive helium–neon laser for phase adjustment, avoiding the influence of the exposure beam on the exposed area. In 2009, the Grating and Measurement Laboratory of Tsinghua University proposed a mosaic exposure method based on latent grating fringe locking to fabricate large-size optical mosaic gratings [182]. The exposure system is shown in Figure 8b. The latent grating generated by the first exposure was used as the reference grating to adjust the substrate posture and phase, avoiding the drift error between the exposure light source and the adjustment light source. The laboratory used this mosaic method to produce a series of 1 × 4 and 2 × 2 optical mosaic gratings with a size of about 100 mm. Among them, the diffraction wavefront peak-to-valley value of the 2 × 2 mosaic grating with a size of (60 + 28) mm× (53 + 30) mm was 0.06λ [187].

The diffraction wavefront quality of the grating fabricated by the mosaic exposure method is high, and it can be extended to the fabrication of large-size gratings of meter-scale. However, there are gap seams or overlapping seams between adjacent exposure areas of the mosaic grating [188], and the seams have an adverse effect on the performance of the grating [189]. Therefore, in subsequent research, it is necessary to optimize the mosaic method to solve the seam problem between adjacent exposure areas.

### 5.4. Scanning Exposure Lithography

Scanning exposure lithography is a new fabrication technology for large-size gratings. Its principle is to use the exposure interference field formed by two coherent light beams to continuously expose the moving substrate, and use the fringe-locking technology to keep the exposure interference field and the substrate relatively stationary. In 2002, MIT developed a scanning exposure lithography device. The principle of the system is shown in Figure 8c [183,190]. This method used the exposure interference field formed by two thin laser beams with an aperture of 1 mm to expose the moving substrate, so it is also called thin-beam scanning exposure lithography. The two-dimensional precision displacement stage drove the substrate to step in the X direction and scan in the Y direction. Through this scanning–stepping method, large-size gratings could be produced. To lock the phase of the exposure interference fringes, they used a beam splitter to generate a heterodyne signal, which was used to measure and adjust the phase difference between the two laser beams. A laser interferometer was installed on one side of the substrate displacement stage to measure the posture and position errors of the stage and feed them back to the compensation system. In 2008, the scanning exposure lithography machine Nanoruler II was launched by the joint team of MIT and PGL. They produced a large-size grating with a size of 910 mm × 420 mm and a grating line density of 1740 lines/mm for Japan’s LEFX project. Its diffraction wavefront error was less than λ/3 @ 632 nm and its diffraction efficiency was greater than 95% [191]. In recent years, the Changchun Institute of Optics and Fine Mechanics has studied the key technologies of scanning exposure lithography, such as the phase-locking of exposure interference field fringes, fringes measurement and adjustment methods, exposure beam quality control methods, and grating diffraction wavefront error control methods [192,193,194,195]. The advantage of the thin-beam scanning exposure method is that the thin beam is directly used to scan and expose the substrate without the beam expansion and collimation elements, so the quality of the exposure interference field is better [196]. However, this method requires very precise control of the exposure interference field and the substrate, which complicates the system. Moreover, to reduce the diffraction wavefront error, the exposure system and the precision measurement system require very high environmental stability.

Based on the thin-beam scanning exposure lithography, the Grating and Measurement Laboratory of Tsinghua University proposed a broad-beam scanning exposure method [197]. The exposure interference field width was 10 mm. A displacement stage was used to drive the substrate to move continuously at a uniform speed in the direction perpendicular to the grating lines. The reference grating was placed next to the substrate. During exposure, the reference fringes generated by the reference grating were used to lock the phase of the exposure interference field and the substrate posture, so that the exposure interference fringes remained stationary relative to the moving substrate. The laboratory fabricated a 40 mm × 40 mm low-stray-light grating through this scanning exposure method. Since this scanning exposure method relies on the reference grating for phase-locking of the interference field, and the size of the reference grating determines the maximum processing size, the expansion to larger gratings is limited. In 2017, the laboratory proposed a new self-reference scanning exposure method [184]. The system is shown in Figure 8d. They first statically exposed a part of the substrate to produce a latent grating for fringe-locking during scanning exposure. As the scanning proceeds, new latent gratings were continuously generated and replaced the old latent gratings in subsequent scanning exposure, which theoretically allowed the length of the grating to be extended indefinitely. By this method, the laboratory has produced a 200 mm × 100 mm diffraction grating with a peak-to-valley value of the diffraction wavefront error as low as 0.065λ. Compared with the thin-beam scanning exposure method, the broad-beam scanning exposure method has higher processing efficiency and lower control difficulty, and has the ability to fabricate meter-scale gratings. In 2025, Li et al. from CIOMP used the scanning exposure method to fabricate a large-sized grating with a groove line density of 1740 lines/mm and an area of 1500 mm × 420 mm [198]. The wavefront aberration reached 0.327λ @ 632.8 nm and the wavefront gradient reach 16.444 nm/cm.

## 6. Conclusions and Prospects

At present, the fabrication technology of large-size diffraction gratings is mainly divided into grating tiling technology, grating ruling technology, and laser interference lithography. Grating tiling technology has the advantages of high efficiency and low cost, and is widely used in astronomical telescopes and chirped-pulse amplification systems. However, in practical applications, grating tiling technology has high requirements on the accuracy and stability of the grating pose error detection and alignment system, and there are also some problems such as spectral energy loss. Therefore, the real-time detection and rapid alignment of grating tiling errors and the long-term stability of tiled gratings will still be the focus of future research.

Grating ruling technology relies on high-precision grating ruling engines, which can process special grooves, and fabricate the gratings with high aspect ratios. However, the processing efficiency of grating ruling engines is very low, and currently it is impossible to fabricate meter-scale gratings. During continuous ruling process, the wear of diamond tools will reduce the ruling accuracy. Therefore, in future research, researchers should focus on improving the processing efficiency of ruling engines, improving the wear resistance of ruling tools, and expanding the maximum processing size.

Laser interference lithography is mainly divided into single-exposure lithography, mosaic exposure lithography, and scanning exposure lithography. The grating quality and processing efficiency of single exposure are relatively high, but it is difficult to expand the processing size due to the limitation of the collimating lens aperture. Mosaic exposure lithography currently still faces many problems in the efficient manufacturing of large-size gratings due to the limitation of the reference grating size, and the mosaic gratings have harmful seams. Scanning exposure lithography can fabricate high-accuracy meter-scale gratings, but it has high requirements on environmental stability and for the measurement and feedback control systems, making the system relatively complex. In recent years, LIL has developed rapidly, especially scanning exposure lithography, which has broken through the limitations of lens aperture and exposure field area, and successfully produced single meter-scale gratings. With the further development of measurement and control technology, scanning exposure lithography will be the most advantageous for the fabrication of single large-size gratings.

## Figures and Tables

**Table 1:** The advantages and disadvantages of main fabrication technologies of diffraction gratings.

Grating Fabrication Tech	Advantages	Disadvantages
Mechanical ruling	Suitable for low groove-density gratings and blazed gratings.	Ghost lines and stray light, low processing efficiency.
Electron beam lithography	Very high resolution.	Very low processing efficiency.
Projection lithography	High efficiency and resolution.	Has difficulty processing heavy substrates.
Nanoimprint lithography	High efficiency and resolution for small scale manufacturing.	Worn imprint molds reduce accuracy.
Laser interference lithography	High efficiency, no ghost lines.	Has difficulty processing complex patterns.

**Table 2:** The advantages and disadvantages of three types of ruling engines.

The Type of Ruling Engines	Advantages	Disadvantages	Applicable Scenarios
Continuous	High positioning accuracy of the groove lines. High ruling efficiency.	Low quality and linearity of groove lines.	Large-size gratings.
Intermittent	High quality of groove lines.	Difficult positioning and low ruling efficiency.	High line-density gratings. Variable period gratings.
Stop-and-go	Combining the advantages of the other two types,	The PZT is easily damaged because of frequent use, and is difficult to control.	Large-size gratings. High line-density gratings.

## Data Availability

The data presented in this study are available on request from the corresponding author.
